# Cell communication and signaling: how to turn bad language into positive one

**DOI:** 10.1186/s13046-019-1122-2

**Published:** 2019-03-13

**Authors:** Claudia Chiodoni, Maria Teresa Di Martino, Francesca Zazzeroni, Michele Caraglia, Massimo Donadelli, Stefania Meschini, Carlo Leonetti, Katia Scotlandi

**Affiliations:** 1 Molecular Immunology Unit, Department of Research, Fondazione IRCCS Istituto Nazionale dei Tumori, Milan, Italy; 2 Department of Experimental and Clinical Medicine, University of Catanzaro “Magna Graecia”, Catanzaro, Italy; 3 Department of Biotechnological and Applied Clinical Sciences, University of L’Aquila, L’Aquila, Italy; 4 Department of Biochemistry, Biophysics and General Pathology, University of Campania “L. Vanvitelli”, Naples, Italy; 5 Department of Neurosciences, Biomedicine and Movement Sciences, Section of Biochemistry, University of Verona, Verona, Italy; 6 National Center for Drug Research and Evaluation, National Institute of Health, Rome, Italy; 7 UOSD SAFU, IRCCS-Regina Elena National Cancer Institute, Rome, Italy; 8 Experimental Oncology Lab, CRS Development of Biomolecular Therapies, Orthopaedic Rizzoli Institute, Bologna, Italy

**Keywords:** microRNAs, Extracellular vesicles, Exosomes, Tumor microenvironment, Cancer therapy

## Abstract

Cell-to-cell communication has a critical role during tumor development and progression, allowing cancer cell to re-program not only the surrounding tumor microenvironment, but also cells located at distant sites. The crosstalk between neoplastic cells and accessory elements, such as immune and stromal cells, fosters several processes that are necessary for tumor progression and dissemination, such as angiogenesis, immune-escape, epithelial-to-mesenchymal transition, invasion and multi-drug resistance. There are several means by which cells communicate to each other, either by direct cell interactions through membrane receptors and ligands, or by releasing soluble molecules, such as growth factors, cytokines and chemokines. More recently, additional means of cell communication have been identified, such as microRNAs and extracellular vesicles. These two peculiar ways of cell-to-cell interaction were the focus of the 31st Annual Conference of the Italian Association of Cell Cultures (AICC).

## Presentation of the conference

The 31st Annual Conference of the Italian Association of Cell Cultures (AICC), entitled “Cell Communication and Signaling: how to turn bad language into positive one” was held in Bologna, at the Rizzoli Orthopaedic Institute on November 27th and 28th, 2018. Katia Scotlandi, President of AICC, from the Rizzoli Orthopaedic Institute, and Alessandra Care’ from the Istituto Superiore di Sanità in Rome, were the scientific coordinators of the meeting.

This year the annual conference was focused on the different means of cellular communication in cancer, such as microRNAs (miRNA) and extracellular vesicles, and on the possibility of taking advantage of these signals for diagnostic and therapeutic purposes. Indeed, while the relevance of these means of communications in fostering tumor growth and dissemination by affecting distant cellular compartments and rendering cancer a systemic disease is established by a plethora of studies, growing evidence is showing that these signals can be exploited for therapeutic approaches. The conference also covered the recently developed techniques of 3D cultures and organoids as systems capable of reproducing, at least in part, the complexity of an organ or of a tumor that can be employed as new tool for biomedical research and application. The meeting was organized in 2 days with four thematic sessions, an opening and a closing lecture, with the contributions of national and international speakers. An additional session was dedicated to the exhibitors’ presentation of new technologies and instruments for exosome isolation, gene expression profiling from ultra-low sample input, label-free live cell imaging and real-time live cell metabolic analysis.

## Opening lecture

The AICC conference was opened by the lecture of George Calin, from The University of Texas, MD Anderson Cancer Center, in Houston (TX, USA). His lecture, entitled “About Noam Chomsky, genomic immunity, non-coding RNAs and diseases”, provided an overview of his outstanding studies on non-coding RNAs, from the findings of miRNAs involved in cancer to the evidence of the presence of xeno-miRNAs in humans and the discovery of transcribed piknons. Along his talk, Calin gave a “semiotic” view of the genome, which, as a sentence, is based on patterns that can be sequences of nucleic acids or amino acids, instead of alphabet letters. The non-coding RNA “dictionary” of the human genome represents a relevant part of it, as most of the non-coding RNAs have regulatory functions [1]. In the early 2000’s Calin, when working as post-doc in Carlo Croce’s lab, significantly contributed to define the relevance of miRNAs, identifying the frequent deletion or down-regulation of miR-15 and -16 in chronic lymphocytic leukemia (CLL) patients and providing the first evidence for the involvement of miRNAs in human tumors [2, 3]. As a clear example of the contribution of miRNAs in human cancers, Calin showed his study on miR-155, in which his group has identified the link between this miRNA with the tumor suppressor p53 and the role in therapy resistance [4]. MiR-155 is considered an oncogenic miRNA and is indeed up-regulated in many human cancers, including solid tumors and hematological malignancies. Its expression is increased in more aggressive tumors and correlates with therapy resistance. However, until Calin’s study, the molecular mechanisms responsible for miR-155-induced therapy resistance were not clear. By using in vitro tumor cell lines they firstly confirmed the association between miR-155 expression and the resistance to chemotherapeutic agents commonly used to treat patients and then they identified a miR-155/p53-negative feedback loop, involved in chemo-resistance, by which p53 inhibits the expression of miR-155 through the direct binding in the downstream region of miR-155 that, in turn, binds to the 3′untranslated regions (UTR) of *TP53* gene. Additionally, Calin showed that the targeting of miR-155 could serve as adjuvant to standard of care chemotherapy in a lung cancer in vivo model [4]. Such preclinical results paved the way for clinical trials testing the safety, tolerability and pharmacokinetics of LNA-based anti-miR-155 MRG-106 in patients with mycosis fungoides, CLL, diffuse large B-cell lymphoma or adult T-cell leukemia/lymphoma.

Over the past decade, the crosstalk between tumor microenvironment and cancer has been largely explored, as previously discussed in the 30th annual conference of AICC at Fondazione IRCCS Istituto Nazionale Tumori (Milan, Italy) in 2017 [5]. In this context, Calin highlighted the functional association between cancer development and circulating small and long non-coding RNAs. He actively contributed to the finding of “foreign” miRNAs encoded by non-human genomes (so-called xeno-miRNAs), such as viral miRNAs, in human body fluids that can be used as biomarkers [6]. Indeed, he showed data on the differential expression of viral miRNAs in the plasma of patients early post-surgery and in sepsis in comparison with healthy volunteers and their functional involvement in sepsis acting as agonist of TLR8 in a positive feedback that may lead to cytokine dysregulation [7]. The measurement of viral miRNAs by qPCR has the potential to become the gold-standard method to detect certain occult viral infections in clinical practice, as demonstrated for Kaposi sarcoma herpes virus (KSHV) using independent multi-institutional cohorts of plasma samples [7, 8]. Calin then reported recent finding on piknons, non-random pattern of repeated elements frequently found in the 3′-UTR of genes of the human genome [9]. Performing multivariate analyses of data from colorectal cancer patients, Calin and colleagues found that N-BLR, a primate-specific long non-coding RNA, facilitates migration primarily via crosstalk with E-cadherin and ZEB1. They showed that this crosstalk is mediated by a pyknon, a short ~ 20 nucleotide-long DNA motif contained in the N-BLR transcript and is targeted by members of the miR-200 family [10]. By using a microarray approach they found multiple such loci that are differentially transcribed between healthy and cancer tissues, identifying several new loci whose expression correlates with the colorectal cancer patients’ overall survival [10]. In the conclusion of his lecture on the evolutionary medicine in tumor biology, Calin introduced the concept of genomic immunity in which pervasive non-coding RNAs, such as pyknons, provide genomic protection. When this finely-tuned system is malfunctioning, because of an excess of viral miRNAs or a reduction in the pyknons levels, a pathological condition, such as viral infection, sepsis, cancer or auto-immune disease, can occur.

### Session 1. CIRCULATING SIGNALS AND CANCER

The advent of genomic-based precision medicine led to the implementation of biomarker testing in cancer. Biomarkers are the key to personalized treatment in cancer patients. In recent years, much of the research on blood biomarkers in cancer has shifted from protein-based to nucleic acid-based molecules. DNA and RNA, like proteins, are released from tumors into the bloodstream. Compared to tissue-based biomarker analysis, that still represents the gold standard, the use of cell-free nucleic acids allows minimal invasive access and ease of serial monitoring, in particular when tumor tissue is not available, or it is insufficient for molecular testing. To date, the use of liquid biopsy refers to those tests performed on body fluids aiming to determine prognosis and predict responses to therapy. Although in most situations biomarkers tend to increase with disease progression and decrease with regression, paradoxical increases, known as spikes or surges, can occur after the commencement of chemotherapy, especially in patients with extensive metastatic burden. These transient increases are usually not related to tumor progression but appear to be the result from therapy-induced apoptosis or necrosis of tumor cells.

**Fig. 1:**
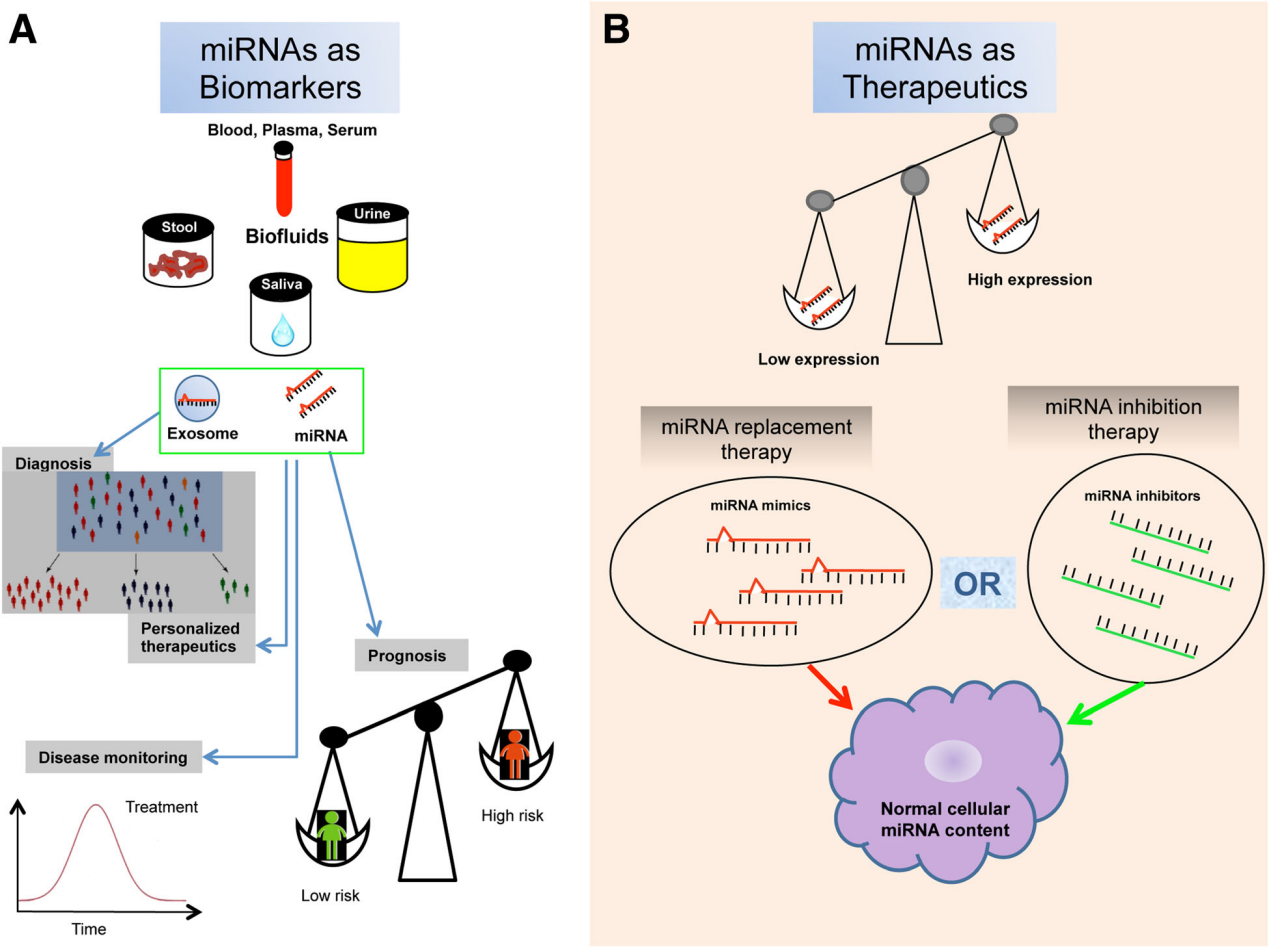
The utility of miRNAs in diagnosis and therapy. miRNAs can be harvested from biological fluids and used as liquid biopsies, in which free circulating miRNAs or exosomal miRNAs can be captured and analyzed ex vivo (**a**). They have proved useful biomarkers for diagnosis, prognosis, personalized therapies or disease monitoring. miRNAs can also be employed as therapeutics or therapeutic targets: miRNA mimics can be used for replacement therapies by the use of delivery systems while miRNA inhibitors, such as locked nucleic acid-antisense oligonucleotides, for inhibition approaches (**b**)

Circulating miRNAs turned out to be good biomarkers for cancers because they regulate gene expression and are involved in cancer initiation and development (Fig. 1a). In addition, miRNAs are easy to detect, and sampling blood or serum is non-invasive and simple to implement. Nevertheless, the definition of reproducible and reliable biomarkers to be applied in clinical practice is still far. miRNA expression levels in biofluids are affected by a range of pre-analytical and analytical challenges in experimental design, from sample collection to data analysis, all contributing to the extremely poor agreement between the circulating miRNA signatures reported by different research groups. In this context Gabriella Sozzi from Fondazione IRCCS Istituto Nazionale dei Tumori in Milan (Italy), reported data from her Institution by the use of a liquid biopsy approach that brought to the development of a minimally invasive molecular test, based on miRNA evaluation in plasma, useful for risk assessment and early detection of Lung cancer (LC). They identified a plasma miRNA signature (miRNA Signature Classifier, MSC) composed of 24 miRNAs which showed high performance in terms of sensitivity and specificity in subjects enrolled in two independent screening trials for LC with a combined low-dose computed tomography (LDCT) [11]. These results prompted the researchers to launch in 2013 a unique prospective trial, called bioMILD (http://www.biomild.org), to test the efficiency of a LDCT-MSC approach as first-line screening tests in a large cohort of 4000 smokers, 50 years or older. In the study design, the combination of the results from the two tests determines the subsequent diagnostic course. Interestingly, the MSC was developed using plasma samples collected longitudinally along the trial and the classifier was able to identify a risk profile to develop lung cancer up to 2 years before a significant tumor burden was visible [12].

This observation led the researchers to hypothesize that such circulating miRNAs could be released not merely by cancer cells but rather by the damaged lung microenvironment and the host immune cells that may sustain tumor development. Thus, such signals could represent very early changes reflecting the biological reaction of the host to cancer development. Sozzi’s group recently focused the attention in understanding the source and contribution of different cell types to the pool of circulating miRNAs using both in vitro models and clinical samples. They observed a cell-type specific expression and topography of several miRNAs in lung normal and cancer tissues and changes of miRNA levels during phenotype modulation of immune and stromal cells, consistent with deregulation of the same miRNAs in plasma of LC subjects. Since miR-320 was the miRNA most overexpressed in activated neutrophils and in agreement with its high level in the plasma of MSC-positive subjects, the authors focused on this miRNA for further studies. They found that miR-320 levels were significantly associated to polymorphonuclear (PMN) cells count in the blood of lung cancer screening patients and its expression levels were higher in neutrophils isolated from MSC-positive subjects, either in cells and in the culture medium, as well as in exosomes. Moreover, the authors showed that miR-320 secreted by PMNs is shuttled into macrophages promoting M2 pro-tumorigenic phenotype through STAT4 down-modulation, thus proving that circulating miRNAs may act in paracrine signaling and have a functional role in lung carcinogenesis and immunosuppression [13]. At the end of her talk, Gabriella Sozzi showed the possible utility of MSC in LC immunotherapy settings. Using a cohort of 140 consecutive advanced NSCLC patients treated with immune checkpoint inhibitors they found that the combination of plasma MSC risk level and PD-L1 expression in the tumor was associated with patients survival [14]. The conclusion of Gabriella Sozzi supports the hypothesis that plasma MSC, reflecting an impaired tumor immune contexture, could complement PD-L1 tumor expression for the identification of a subgroup of patients who do not benefit from immunotherapy.

MiRNA are broadly deregulated in many types of tumor where they affect all the hallmarks of cancer, sustaining proliferative signaling, evading growth suppressors, resisting to cell death, activating invasion and metastasis, and inducing angiogenesis. An increasing number of researches identified miRNAs not only as potential biomarkers for diagnosis or prognosis of human cancer, but also as therapeutic targets or tools [15] (Fig. 1b). In this context, Massimo Negrini from University of Ferrara (Italy) presented a hepatocellular carcinoma (HCC) model for miRNA therapeutic investigation. Negrini’s group developed a transgenic (TG) mouse model carrying a liver-deregulated miR-221 (TG221), characterized by the occurrence of spontaneous nodular lesions in the liver of approximately 50% of male mice and by a strong acceleration of tumor development in mice treated with diethylnitrosamine (DENA). This animal model has been used to investigate the role of two miRNAs that are deeply deregulated in human hepatocarcinoma. First, they investigated the therapeutic activity of an anti-miRNA oligonucleotide (AMO) against miR-221 in TG221 mice and observed a significant reduction in the number and size of lesions in anti-miR-221-treated mice compared to mice treated with DENA only, together with a persistent decrease of miR-221 levels in liver of the AMO-treated mice [16]. The TG221 HCC mouse model was also employed for a miRNA replacement approach, to restore miR-199 expression that is down-regulated in human HCC, using a miR-199a-3p unmodified single-stranded RNA oligonucleotide. The in vivo delivery of the miR-199a-3p mimic was facilitated by the use of lipid nanoparticles as vehicle. The antitumor activity in TG221 mice was evaluated in comparison with a miRNA scrambled control and sorafenib tosylate treatments. Administration of miR-199a-3p mimics in the TG221 transgenic mouse bearing liver cancer led to a significant reduction in both number and size of tumor nodules compared to control animals. Further analysis confirmed in the liver of treated mice a down-regulation of the miR-199a-3p direct targets, such as mammalian target of rapamycin (mTOR) and p21 activated kinase 4 (PAK4), ultimately leading to the repression of FOXM1 [17]. Remarkably, the anti-tumor activity of miR-199a-3p mimic was comparable to that obtained with sorafenib. These results suggest that miR-199a-3p may be considered a promising HCC therapeutic option. Interestingly, the TG221 mouse treated with carbon tetrachloride was found to recapitulate the fibrosis-cirrhosis-HCC natural history seen in human. In this experimental setting, the model was used to evaluate if miRNA-based therapies can prevent liver cancer. The data presented by Massimo Negrini indicate that anti-miR-221 or miR-199 mimics prophylaxis is able to reduce nodule size and malignant potential (Shankaraja et al., unpublished data), suggesting that tumor prevention could represent the setting in which these molecules could work best. However, they also indicate that re-expression of multiple miRNAs is needed to fully prevent the appearance of tumors in this mouse model. The natural history of HCC development suggests the possibility of applying miRNAs prophylaxis protocols.

### Session 2. Tumor-microenvironment interactions

Cell communication has a fundamental role in allowing cancer cell to coopt and modulate stromal and immune cells. The active crosstalk between the tumor and the surrounding cells fosters angiogenesis, immune-escape, epithelial-to-mesenchymal transition, formation of a pre-metastatic niche, metastases and multi-drug resistance. Cell communication can occur either through direct cell interactions or by the release of soluble factors, such as cytokines, growth factors and chemokines. A noteworthy way of communication between tumor cells and the tumor microenvironment (TME) is represented by extracellular vesicles (EVs).

**Fig. 2:**
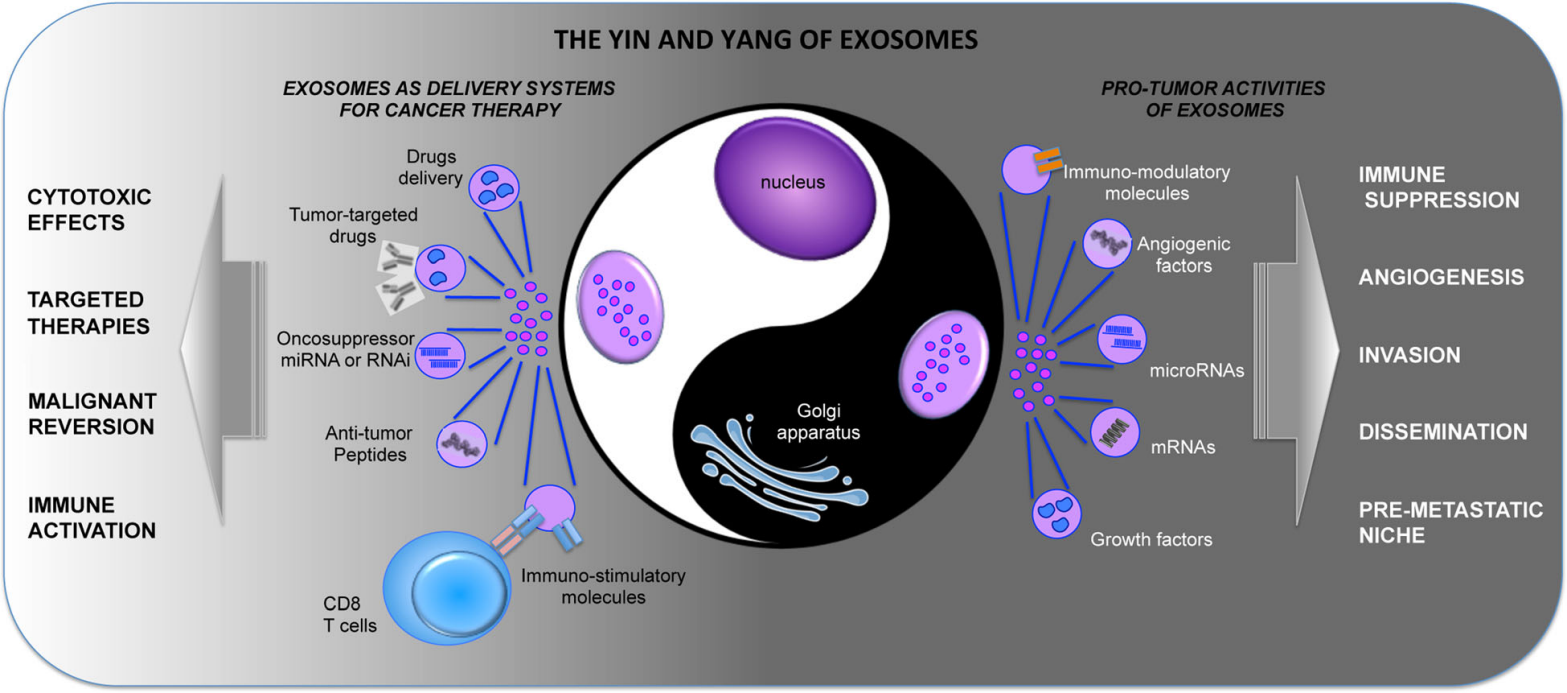
The Yin and Yang of exosomes. Exosomes are released by a variety of cell types, including cancerous cells. In this context, exosomes contribute to tumor progression and dissemination. Indeed, they carry malignant information in form of nucleic acids, either mRNAs or miRNAs, or proteins, such as growth factors, cytokines, chemokines, or angiogenic and immuno-regulatory molecules. Through their cargo cancer-derived exosomes can foster different aspects of tumor growth, as neo-vascularization, immune suppression, invasion, dissemination and pre-metastatic niche formation. On the other hand, exosomes, in light of their structure, can be considered as a new generation of a natural nanoscale delivery systems and exploited for therapeutic approaches. Specific cell types can be genetically modified and used as cell factories for the production of armed exosomes, for example carrying onco-suppressor miRNAs, or shRNA, or targeted exosomes, carrying receptors or antibodies for binding to specific cell types. Furthermore, the exosome liposome-like structure allows them to be loaded with various drugs

EVs are circular fragments of membranes that carry various bioactive molecules including membrane receptors, proteins, mRNAs, microRNAs, and organelles, thus being able to stimulate target cells by receptor-to-ligand interaction or by transferring their cargo. The most studied EVs are the exosomes, which are derived from a process of fusion between intracellular organelles (i.e. endosomes and lysosomes) and plasma membrane as well. Exosomes released from cancerous cells, with their cargo of growth factors, cytokines, angiogenic molecules, miRNAs, and immunosuppressive factors, actively contribute to tumor progression and dissemination (Fig. 2).

The first presentation in the session was given by Stefano Fais, from Istituto Superiore di Sanità, in Rome (Italy), who focused on the effects of tumor acidity on exosome release from tumor cells. He started from previous observations by his group: tumors are acidic, because neoplastic cells are able to generate energy by non-oxidative breakdown of glucose, and proton pump inhibitors are able to rescue tumor acidity to normal pH levels, both in vitro and in vivo, thus opening a route for a therapeutic use of these inhibitors in association with chemotherapy in various refractory tumors [18, 19]. Notably, Stefano Fais has recently demonstrated that tumor low pH has an impact on exosome release and trafficking. His group investigated the role of tumor pH in regulating exosome release from tumor cells in the context of prostate cancer (PCa) by performing both Nanoparticle Tracking Analysis (NTA) and nanoscale flow-cytometry [20]. In vitro, when prostatic cancer cells were cultured in acidic medium, an increased release of nanovesicles, positive for both PSA and the exosome marker CD81, was observed. Interestingly, in plasma from prostate cancer patients, the concentration of PSA^+^-exosome resulted higher than in healthy donors and subjects with benign prostatic hyperplasia, indicating that tumor microenvironmental acidity has an impact on exosome release in vivo [20]. Moreover, the increased release of exosomes from tumor cells was not a specific feature of PCa but is a common feature of all cancers [21]. Overall, his data indicate that treatment with proton pump inhibitors may dramatically impact on tumor-TME communication, by inhibiting exosome release, and hence may represent a new therapeutic strategy in cancer.

Anna Maria Teti, from University of L’Aquila (Italy), presented data on a fascinating aspect of tumor-TME cross-talk, the interaction between the endosteal niche and tumor dormancy. In the endosteal niche, spindle-shaped N-cadherin+CD45- osteoblastic (SNO) cells, which are located mainly on the surface of cancellous/trabecular bones, are associated with N-cadherin+ stem cells (HSC) and support their growth through specific interaction between N-cadherin and β-catenin [22]. The TME has a key role in determining the ultimate fate of tumor cells, including dormancy of metastatic cancer cells. Late recurrence is particularly relevant in breast cancer and one of the preferred site of breast cancer metastasis is indeed the bone [23]. The study done by Teti’s group aimed at clarifying the mechanisms of breast cancer dormancy in the bone and the role of bone marrow microenvironment in this phenomenon. She demonstrated that SNO cells are cell cycle-arrested, poorly differentiated osteoblasts, which express the Notch2 ligand Jagged1. In in vivo experiments, they observed the engraftment of single cell cycle arrested breast cancer cells close to endosteal SNO cells, thus suggesting a role of the endosteal TME in supporting breast cancer cell dormancy. This hypothesis was confirmed by in vitro experiments showing that breast cancer cells plated on SNO cells reduced their own proliferative rate and expressed high level of Notch2. The involvement of Notch signaling in this context was formally demonstrated by Notch inhibition. In fact, Notch2^high^ breast cancer cells, grown with SNO cells, rescued their proliferative rate in presence of Notch inhibitors and acquired HSC mimicry and stem cell phenotype. Additionally, these cells were resistant to chemotherapy treatment. To complete this picture, Anna Teti showed that Notch2^high^ breast cancer cells generate less bone and liver metastasis in vivo, and that Notch inhibition was able to reactivate dormant breast cancer cells and promote metastatic dissemination to the liver. In agreement with those preclinical results, at the end of her presentation she showed that in breast cancer patients, high expression of Notch2 is correlated with better prognosis, thus potentially representing a new prognostic marker for recurrence.

The last invited speaker of this session, Giovanni Camussi from University of Turin (Italy), highlighted another interesting aspect of tumor-TME interaction regarding the ability of tumor-derived extracellular vesicles (EVs) to trigger angiogenesis and promote the formation of a pre-metastatic niche. He showed that renal carcinoma stem cells, positive for the mesenchymal stem cell marker CD105, were able to stimulate the growth and induce an angiogenic phenotype in normal lung endothelial cells [24]. These effects were due to an epigenetic modification of endothelial cells mediated by cancer stem cells (CSC)-derived EVs. EVs are able, indeed, to transfer miRNAs and RNA-binding proteins from CSC to other cells in the TME. In the context of renal carcinoma, Giovanni Camussi demonstrated an up-regulation of 24 miRNA and a down-regulation of 33 miRNA in CD105^+^ EVs in comparison to CD105^−^ EVs. Many of these miRNAs are known to be associated with tumor invasion and metastasis and have been shown to be deregulated in renal carcinoma and in other tumors. Indeed, he reported that CD105^+^ EVs, but not CD105^−^ EVs, carry mRNAs for genes involved in angiogenesis such as VEGF, FGF2, angiopoietin1, ephrin A3, MMP2 and MMP9. These vesicles also significantly enhanced the expression of VEGFR1, VEGF and MMP2 in lung endothelial cells and of MMP9 in the whole lung in vivo, thus favoring the formation of a lung pre-metastatic niche [24]. CD105^+^ EVs are able to promote phenotypic changes not only in endothelial cells but also in mesenchymal stromal cells (MSCs). Indeed, in vivo experiments in which renal tumor cells were co-injected with EV-stimulated MSCs demonstrated a role for CD105^+^ EVs in supporting tumor development and vascularization [25]. Another key aspect of tumor progression is the ability of cancer cells to down-modulate the immune response. Giovanni Camussi ended his talk showing that CD105^+^ EVs play a role also in this aspect, demonstrating that CD105+ EVs impair maturation of DCs and T cell immune response by a mechanism involving HLA-G [26].

### Session 3. Clinical potential of exosomes

The delivery of drugs or other agents specifically to pathological tissues is one of the challenges of medicine for the treatment of neoplastic and inflammatory diseases. In this context, the use of nanocarriers can be useful in light of the ability of nanoscale materials to trespass the fenestrated vessels of inflammatory and cancer tissues and to accumulate in these tissues because of the lack of lymphatic drainage (the so-called enhanced permeation and retention effect) [27]. In addition, the development of functional nanomaterials is of great importance and significance for the active targeting and re-targeting of the affected tissues. Nevertheless, exogenous nanomaterials have a tendency of inducing undesired immune responses and nano-protein interactions, which may result in toxicity and therapy failure. Exosomes can be considered as a new generation of a natural nanoscale delivery system. Indeed, exosomes secreted by different types of cells carry different signaling molecules (such as RNAs and proteins) and thus have a great potential for targeted drug delivery and therapy [28] (Fig. 2). Furthermore, the exosome liposome-like structure allows them to be loaded with various drugs. Hence, the potential of exosomes in drug delivery, tumor-targeted therapy, and immunotherapy has been widely investigated in recent years [29].

In this session, the clinical perspective on exosome-based therapeutic strategies has been discussed, starting from the description of artificial nanocarriers, niosomes, similar to exosomes for composition and delivery properties, discussed in this session by Maria Carafa, from La Sapienza University of Rome (Italy). Niosomes are non-ionic surfactant-based vesicles [30] that can be considered a novel drug delivery system. The twofold structure of surfactant molecules encourages them to orient in a bilayer architecture. In this two-layered structure, hydrophilic and hydrophobic ends of surfactant tend to arrange into external and internal sites, respectively. The most used surfactants are SPAN, TWEEN and BRIJ [31]. This lamellar morphology allows them to entrap different types of drugs in a unique carrier simultaneously. Several factors can affect the structure of niosomes, such as the nature and type of surfactants, the amount of cholesterol, the critical packing parameter and the drug used [32]. In the end of her presentation, Maria Carafa underscored the fact that nanocarriers are dinamically interacting with other biological entities**,** such as proteins, cell membranes and intracellular compartments, and therefore understanding the fate of nanocarriers is a necessary step for optimizing their design in order to deliver cargo molecules more effectively and with better targeting actions [33, 34].

Niosomes can be designed in order to be administered via different routes, such as oral, topical, and employed for dermal and transdermal delivery, for ocular, oral, lung and parenteral delivery, and used for gene delivery, diagnostics/theranostics and for natural product encapsulation. Although niosomes are similar to liposomes in structure, niosomes are more stable and also more cost-effective than liposomes and remain in the bloodstream for a reasonable time, which is useful for targeted drug delivery.

The second presentation, held by Simona Fontana from the University of Palermo (Italy), focused on the role of tumor-derived exosomes (TDEs) in cancer signalling and progression. Indeed, an important emerging function of exosomes secreted by tumor cells is the ability to influence the biological behavior of other cells, either neoplastic or normal cells not only in the surrounding tumor microenvironment but also, being released in the blood stream, in distant districts. In this context, several pieces of evidence indicate an active role for TDEs in the establishment of the pre-metastatic niche. Simona Fontana presented data on the crosstalk between cellular elements of the bone marrow microenvironment and tumor cells in the context of chronic myeloid leukemia (CML), which affects disease progression. Such crosstalk occurs through soluble factors (cytokines and growth factors) and extracellular matrix components. Angiogenesis is a key step for the progression of CML, and she showed results on the role of exosomes in this process. She showed that the addition of exosomes to human vascular endothelial cells (HUVEC) in vitro induces an increase of ICAM-1 and VCAM-1 cell adhesion molecules and of IL-8 expression [35] and that exosomes released from CML cells stimulate bone marrow stromal cells to produce IL 8 that, in turn, modulates the leukemia cell malignant phenotype [36]. On the same line, but in different tumor histotypes, she showed that TDEs from multiple myeloma and lung cancer cells drive osteoclast differentiation in RAW264.7 macrophagic cells. In the context of lung cancer, she showed that such effect is mediated by amphiregulin contained in NSCLC exosomes that activates the EGFR pathway [37]. Additionally, using two isogenic human colon cancer cell lines, established from the same colon cancer patient, she showed that TDEs derived from metastatic cells are able to induce the phenotypic switch of non-metastatic elements by eliciting the mesenchymal to amoeboid transition [38]. Finally, circulating TDEs can represent useful biomarkers to define the molecular signature of a tumour (liquid biopsy) and tools for therapeutic application as delivery systems for drugs, miRNAs, or immunotherapeutic agents.

### Session 4. 3D-organoids models as new tools for biomedical research and application

In recent years, a high technological development has been achieved through the use of three-dimension (3D) techniques, molded scaffolds and organoids, in order to obtain experimental models better resembling the real biological settings [39]. The reason that prompted researchers to improve cell culture from two-dimension (2D) to 3D lies in the fact that 2D condition does not reproduce tissue architecture and cellular heterogeneity. Organoids are structures composed of different types of self-organized cells to better mimic embryonic and tissue development in vitro. The 3D model has proven to be superior compared to conventional cellular models, as it presents architecture and geometric characteristics of the tissues observed in vivo, thus allowing a better evaluation of the response to drugs are used in translational, personalized and regenerative medicine [40].

Tissue engineering is an innovative new therapeutic strategy based on the combination of cells, suitable biomaterials and growth factors used to improve or replace biological structures. Cells can be isolated from different tissues such as bone marrow, fat and synovia, peripheral blood, placenta, umbilical cord and embryo. Mesenchymal stem cells can be isolated, expanded in vitro and they need to survive without malignant transformation. In recent years, there have been huge technological advances in scaffold development through the production of natural and synthetic polymers. Natural or recombinant polymers, plus anabolic factors and platelet-rich plasma, are used to favor and regulate different cellular processes such as proliferation, differentiation and synthesis of specific tissue matrix molecules [41]. In this contest, Brunella Grigolo from IRCCS Istituto Ortopedico Rizzoli, Bologna (Italy), presented data on a meniscus prototype obtained with the use of the 3D bioprinting technique [42]. The meniscus serves many important biomechanical functions, it contributes to load transmission, shock absorption, nutrition, joint lubrication, and proprioception. Damage to the meniscus can be of different types and origins, like acute tears due to trauma or sport injuries, or chronic tears, most often occurring in elderly people, and degenerative meniscal tears that occur after minimal trauma or stress on the knee. Three dimensional (3D) bioprinting is a technique that uses 3D printing technologies to combine cells, growth factors, and biomaterials within a single “bio-ink”, in order to fabricate structures capable of replicating natural tissue characteristics. Patient data are obtained by digital imaging and processed by specific software. The presented study reported that 3D bioprinter was able to produce a meniscus made by Collagen Type I (size 5 mm) that could be seeded with human mesenchymal stem cells and that could represent the starting point for future developments toward the optimization of implants to replace damaged structures.

Recent advances in human-derived 3D cultures for studies on bone metastasis were nicely presented by Milena Fini from IRCCS Istituto Ortopedico Rizzoli, Bologna, (Italy). She explained the complexity of the interaction between tumor cells that metastasize in the bone inducing destructive osteolytic and/or bone forming osteoblastic lesions and the alteration of bone microenvironment producing factors that stimulate tumor growth [43]. When bone metastases were initially studied, one of the limitations was the complex nature of the bone environment. Up-to-now, there are no commercially available models able to mimic the biological processes occurring in patients. To improve the understanding of these phenomena and the interpretation of the results, Fini’s group obtained an in vitro three-dimensional (3D) bone metastasis model by culturing human breast or prostate cancer cells with human bone tissue isolated from female and male patients, respectively. Gene expression profile, protein levels, histological, immunohistochemical and four dimensional (4D) micro-CT analyses showed a noticeable specificity of breast and prostate cancer cells for bone colonization and ingrowth, thus highlighting the species specific and sex-specific osteotropism [44]. Additionally, using this in vitro model, Fini’s group evaluated also the influence of bone remodeling rate, recognizing major differences in tumor growth and colonization between healthy and osteoporotic status. They showed that the altered bone microenvironment due to osteoporosis stimulates tumor cell colonization indicating that the mechanisms directing breast cancer progression could be influenced also by the endocrine status of the patient [45]. These 3D models will be important for the development of new complex cell systems to study the physiological events that occur when cancer cells meet and interact with the bone environment.

Currently there are very few studies on tissue culture models to mimic metastasis of colorectal cancer (CRC) to liver. Most studies on liver colonization are performed in 2D conventional culture models or xenograft models. The 2D culture models fail to recapitulate the pathophysiological gradients of oxygen, nutrient/waste and drug and the cell-extracellular matrix (ECM) interaction occurring in a physiological in vivo setting. The animal model has the advantage of providing a 3D environment with complex cell-cell and cell-ECM interactions. In this contest, Marco Agostini from University of Padua, Padua (Italy), presented a new method of tissue decellularization. This method is widely used in tissue engineering with the aim of creating an a-cellular scaffold that, once repopulated with autologous cells, is re-implanted in the patient to restore the functions of the damaged tissue [46]. Progresses in decellularization techniques provide novel approaches for isolating patient-derived ECM. Three-dimensional cancer models could be used to investigate CRC pathogenesis, progression and treatment response. Only recently, the decellularization process has been applied to the study of tumor progression. The goal of the study presented by Marco Agostini was to develop an in vitro patient-derived 3D bioactive model of colon cancer liver metastasis (CRLM). As initial step, the attention was focused on reproducing the structural and biochemical microenvironment of CRLM by assessing nuclei depletion and preservation of ECM biochemical and biophysical characteristics. Moreover, immunohistochemical analysis showed that the expression and distribution of key ECM components, such as collagen and glycosaminoglycan, were maintained in the decellularized healthy liver (HL) and colorectal cancer liver metastasis (CRLM) compared to native fresh tissue. A real-time non-invasive technique was used to monitor and analyze cell behavior in the metastatic scaffold. Finally, to evaluate whether the patient-derived decellularized scaffolds retained the biological functionality of the native tissue, Luc-ZsGreen+ HT-29 cells were cultured in CRC and CRLM scaffolds, using their respective healthy samples as controls. The results suggested that the ECM of the different scaffolds influenced the behavior of CRC-derived cells. To evaluate if CRLM scaffold induced a pro-migration stimulus to CRC cells, cells were labeled with an antibody to E-cadherin, which is critical for the formation and maintenance of adhesion of epithelial cell-cell contact and loss of its expression could indicate transition between benign to metastatic tumors. CRC cells showed a specific organotypic reduction of E-cadherin expression only in CRLM recellularized scaffolds, compared to HL and CRC. These data provide important indications on the possible use of detergent-enzymatic methods to effectively decellularize healthy and metastatic tissue, preserving the tissue structure and architecture.

Gina Lisignoli from IRCCS Istituto Ortopedico Rizzoli, Bologna (Italy) addressed the issue of development of an in vitro co-culture model for mesenchymal stem cells and synoviocytes focusing on the need to develop a 3D in vitro osteoarthritic (OA) synovial model for studying the role of synovial cells and of their ECM [47, 48]. Healthy synovial membrane is the soft tissue lining the spaces of diarthrodial joints, tendon sheaths and bursae. Synovitis is characterized by synovial lining hyperplasia, sub-intima fibrosis, stromal vascularization and abundant influx of leukocyte. The synovial membrane is studied in vitro by synovial fibroblasts monolayer or cell line (i.e. K4IM) but these models do not take in consideration the role of synovial macrophages or ECM. Synovial explants have the benefit of native synovial membrane structure, but they show high variability and limited amount of samples. Up to now, there is a lack of in vitro systems that represent the pathological synovial tissue. Thus, the development of engineered synovial tissues may help overcoming these limitations. Intra-articular injections of adipose-derived mesenchymal stem cells (ASC) in osteoarthritis animal models reduced inflammation and macrophage markers by specifically adhering to synovial macrophages. Lisignoli underscored the importance of using specific cells able to recreate a milieu that more closely resembles the characteristics of OA synovial tissue. Moreover, the availability of in vitro cell models (synoviocytes) that better reflect the different degrees of OA synovial inflammation could give the opportunity of testing cells or anti-inflammatory drugs in a well-defined and more predictive milieu. At the end, the development of a 3D engineered in vitro synovial membrane model could better reproduced the physiological characteristics of the native tissue and provide a platform able to mimics structural changes occurring at different degree stages.

## Closing lecture and remarks

In the closing lecture of the meeting, Susanne Gabrielsson from the Karolinska Institute (Stockholm, Sweden) addressed the role of exosomes, extracellular nanovesicles which origin from the endosomal compartment and that can carry immunostimulatory molecules, as potential immunotherapeutics for cancer treatment. After a general introduction on exosomes and on the different technologies currently available for their analysis, Susanne Gabrielsson focused her presentation on the feasibility of using Exo in clinical cancer therapy, and mentioned the few clinical trials performed with dendritic cell (DC)-derived exosomes. She described the model system used in her laboratory to measure the immune response elicited by exosomes obtained from ovalbumin (OVA)-loaded DC, stressing the requirement for the presence of the whole antigen in vivo for an efficient immune response, not obtained when the specific peptide is used [49, 50].

Susanne Gabrielsson presented data on how to improve the immunogenicity of DC-derived exosomes by loading DC with the antigen and the iNKT-cell ligand αGalCer (αGC) [51, 52]. In particular, she showed that Exo-OVA/αGC administered intravenously in C57BL/6 mice were able to decrease tumor growth of B16-OVA tumors, and increased antigen-specific CD8 T-cell tumor infiltration and survival, relative to control mice. Interestingly, the number of injections resulted to be critical for the induction of an efficient immune response, as a double shot was able to elicit a production of antibodies with higher avidity compared to a single injection. This aspect should be considered in view of clinical application of the strategy. She went on presenting details on the mechanisms of the response, showing the independency from the MHC of the exosome [53], the requirement of both T and B cells for the immune response [54] and the possibility of using allogeneic exosomes that are equally effective as syngeneic exosomes in the B16 melanoma model. In conclusion, her results obtained in pre-clinical mouse models suggest the possibility of using exosomes for therapeutic purpose in humans.

In conclusion, the 31st Annual Conference of the Italian Association for Cell Cultures, hosted at the Rizzoli Orthopedic Institute, one of the most important institution in this area of medicine in Italy and in Europe, has seen more than 100 participants. Most of them were young researchers who had the chance to learn on the role of cell communication and signaling in pathological conditions and as means for potential therapeutic vehicles, as well as to share their ideas and knowledge in the field. This has been made possible by brilliant presentations from outstanding invited speakers, selected talks and during the interactive poster sessions.

At the end of the conference, as a well-established tradition for AICC, 5 young researchers received prized for either best posters or oral presentation from Fondazione Berlucchi, the Journal of Experimental and Clinical Cancer Research and AICC. We would like to congratulate with the young recipients of the scientific awards and thank all the attendees of the 31st AICC Annual Conference.

## Abbreviations

CLL: Chronic lymphocytic leukemia
EVs: Extracellular vesicles
KSHV: Kaposi sarcoma herpes virus
LC: Lung cancer
LDCT: Low-dose computed tomography
miRNAs: microRNAs
TME: Tumor microenvironment
UTR: Untranslated region
MSC: miRNA Signature Classifier
NSCLC: Non small cell lung cancer
PMN: Polymorphonuclear
TG: Transgenic
DENA: Diethylnitrosamine
HCC: Hepatocellular carcinoma
AMO: Anti-miRNA oligonucleotide
PCa: Prostate cancer
NTA: Nanoparticle Tracking Analysis
SNO: Spindle-shaped N-cadherin+CD45- osteoblastic
HSC: Hematopoietic stem cells
CSC: Cancer stem cells
TDEs: Tumor-derived exosomes
CML: Chronic myeloid leukemia
2D: Two-dimension
3D: Three-dimension
ECM: Extracellular matrix
CRLM: Colon cancer liver metastasis
HL: Healthy liver
OA: Osteoarthritic
ASC: Adipose-derived mesenchymal stem cells
DC: Dendritic cells
OVA: Ovalbumin
αGC: αGalCer
