# Correction to: Cell communication and signaling: how to turn bad language into positive one

**DOI:** 10.1186/s13046-019-1431-5

**Published:** 2019-10-25

**Authors:** Claudia Chiodoni, Maria Teresa Di Martino, Francesca Zazzeroni, Michele Caraglia, Massimo Donadelli, Stefania Meschini, Carlo Leonetti, Katia Scotlandi

**Affiliations:** 1 Molecular Immunology Unit, Department of Research, Fondazione IRCCS Istituto Nazionale dei Tumori, Milan, Italy; 2 Department of Experimental and Clinical Medicine, University of Catanzaro “Magna Graecia”, Catanzaro, Italy; 3 Department of Biotechnological and Applied Clinical Sciences, University of L’Aquila, L’Aquila, Italy; 4 Department of Biochemistry, Biophysics and General Pathology, University of Campania “L. Vanvitelli”, Naples, Italy; 5 Department of Neurosciences, Biomedicine and Movement Sciences, Section of Biochemistry, University of Verona, Verona, Italy; 6 National Center for Drug Research and Evaluation, National Institute of Health, Rome, Italy; 7 UOSD SAFU, IRCCS-Regina Elena National Cancer Institute, Rome, Italy; 8 Experimental Oncology Lab, CRS Development of Biomolecular Therapies, IRCCS Istituto Ortopedico Rizzoli, Bologna, Italy

**Correction to: J Exp Clin Cancer Res**

**https://doi.org/10.1186/s13046-019-1122-2**

In the original publication of this article, [1] the affiliation for Katia Scotlandi needs to be revised, because the author prefers using its Italian name: Experimental Oncology Lab, CRS Development of Biomolecular Therapies, IRCCS Istituto Ortopedico Rizzoli. We apologize for any confusion this may have caused.
